# IntegromeDB: an integrated system and biological search engine

**DOI:** 10.1186/1471-2164-13-35

**Published:** 2012-01-19

**Authors:** Michael Baitaluk, Sergey Kozhenkov, Yulia Dubinina, Julia Ponomarenko

**Affiliations:** 1 San Diego Supercomputer Center, University of California San Diego, 9500 Gilman Drive, La Jolla, CA, 92093, USA; 2 Skaggs School of Pharmacy and Pharmaceutical Sciences, University of California San Diego, 9500 Gilman Drive, La Jolla, CA, 92093, USA

**Keywords:** data integration, search engine, biological ontologies

## Abstract

**Background:**

With the growth of biological data in volume and heterogeneity, web search engines become key tools for researchers. However, general-purpose search engines are not specialized for the search of biological data.

**Description:**

Here, we present an approach at developing a biological web search engine based on the Semantic Web technologies and demonstrate its implementation for retrieving gene- and protein-centered knowledge. The engine is available at http://www.integromedb.org.

**Conclusions:**

The IntegromeDB search engine allows scanning data on gene regulation, gene expression, protein-protein interactions, pathways, metagenomics, mutations, diseases, and other gene- and protein-related data that are automatically retrieved from publicly available databases and web pages using biological ontologies. To perfect the resource design and usability, we welcome and encourage community feedback.

## Background

Diverse web resources and databases, such as UniProt [1], GenBank [2], GeneCards [3], RGD [4], MGD [5], and many others, catalog various properties of genes, ranging from their mapped chromosomal coordinates to the enzymatic function of the proteins they encode. Scientists have to visit many of these databases or web sites for each gene in their candidate gene list; they must learn and remember how to navigate various web sites, each of which accepts different sets of gene identifiers (Entrez Gene, Ensembl, Refseq, UniGene, and other), thus making the navigation difficult and time-consuming. Although these resources are highly informative individually, the collection of available content has more power, if provided in an integrated, unified, centralized context indexed in a robust manner. Although currently there is no single resource that completely describes everything that a researcher might want to learn about a specific gene, a few integrative approaches towards this goal have been developed and include BioGPS [6], Ondex [7], NIF [8], and several databases for cataloging web resources, such as PathGuide [9] and MouseBook [10]. The integrated and comprehensive use of biological information is hampered by the large number of available databases and their fragmentation. ID/naming problems and conflicts in biological data pose additional obstacles towards comprehensive biological data integration. Because of the diverse history of these databases and resources, integration with commonly used molecular database resources, such as NCBI's Entrez [11], is done on a case-by-case basis. At the same time, new online resources are continually being developed and staying abreast of these tools and evaluating their utility is a time-consuming and recurring task.

Web search engines, such as Google, excel at finding a needle in a haystack: a single fact, a single definitive web page. Often, however, the user's objective is not to find a single fact, but to explore all available data concerning a specific genes or proteins. For example, a person diagnosed with diabetes might want to learn all known scientific data about genes associated with this disease. Or, for a researcher who wants to learn 'all about p53 gene/protein', it is much easier to get the information centralized in one place, instead of searching the whole Internet for this information. Even if the researcher knows exactly what to look for, for example, specific post-translational modifications in p53 protein that result in different types of cancer, he/she still might need to browse many different web resources in search of this information.

Here, we propose a solution to the problem of searching biological data, specifically gene/protein-centered data, via semantic integration of data from a broad collection of molecular biology resources and presentation of these data on one web page for each searchable gene/protein. Data have been automatically integrated from more than a thousand databases listed in the Nucleic Acid Research database list [12], as well as from PubMed, Wikipedia, and millions of public web sites that are referenced from these databases. The presented search engine, http://www.integromedb.org, has been designed for browsing available data on gene regulation, gene expression, protein-protein interactions, pathways, metagenomics, mutations, diseases, and many other gene- and protein-related data. IntegromeDB web page is linked to BiologicalNetworks http://www.biologicalnetworks.org[13], which is a Java web-start application designed for in-depth analysis of biological data, building and visualization of gene regulatory and protein-protein interaction networks; the presented web resource and BiologicalNetworks both rely on the same integrated database [14]. Application Programming Interface (API) available at http://integromedb.org/api.jsp provides access to the most features available through the web application and can be used by third-party users and resources. The batch search and retrieval options are also provided at the IntegromeDB web site, http://www.integromedb.org.

## Construction and content

### Data Integration

**Figure 1:**
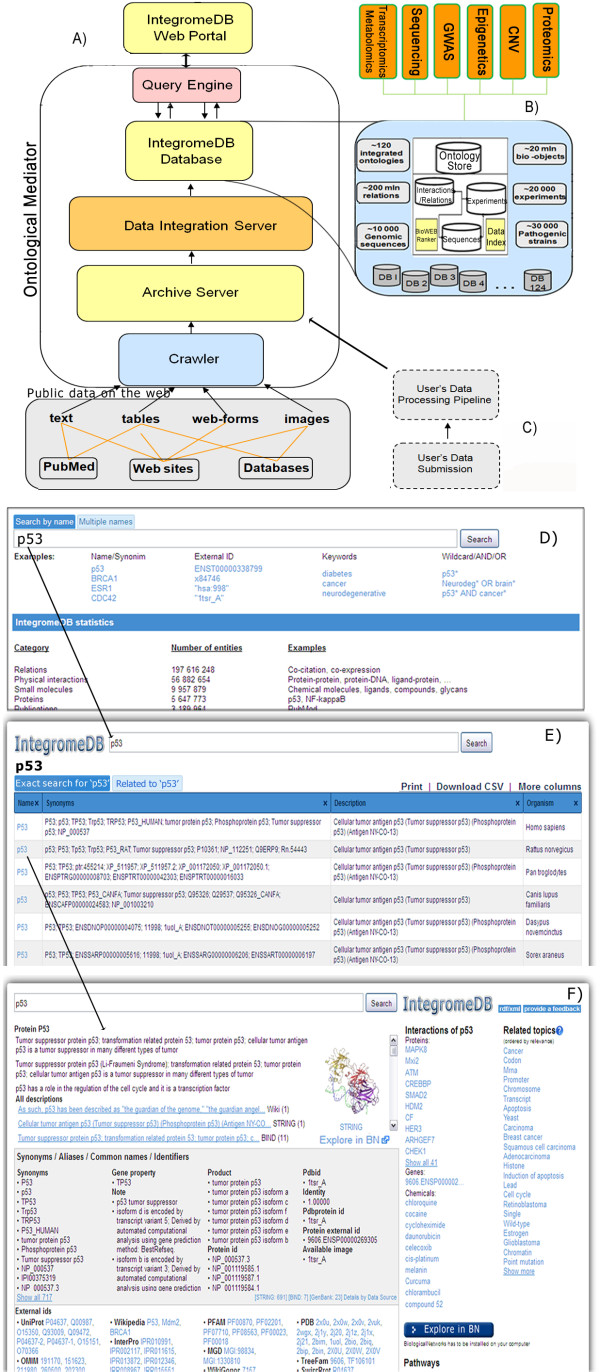
**IntegromeDB architecture. A) **Major architectural blocks of the IntegromeDB system. **B) **Internal database of the IntegromeDB; described in detail in [14]. **C) **The pipeline processing the user's data. **D) **IntegromeDB web page with search examples and search options. For a batch search, input can be uploaded as a text file or by pasting the gene/protein list into a text box. **E) **Search results sorted by relevance score. Clicking the gene/protein name takes the user to the respective gene/protein report page **(F) **(see Figure 2).

**Figure 2:**
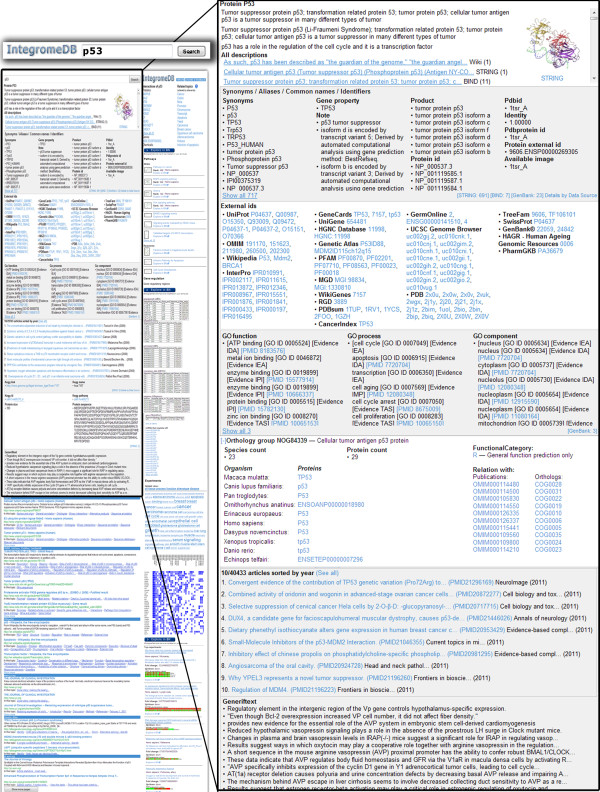
**IntegromeDB gene/protein report page**. Gene/protein attributes (descriptions, synonyms, external ids, GO annotations, orthology, publications, and others) from a variety of data sources are ordered by relevance and clustered by data source.

The basic anatomy of the IntegromeDB data integration system that is behind the presented search engine is described in detail in [14]. Briefly, IntegromeDB is a semantic, graph based, 'deep web' data integration system that automatically captures, integrates, and manages publicly available data related to functioning of genes and proteins (Figure 1; see also Figure S1 in Additional file 1). The system's internal database is structured as a meta-graph database that contains the features attributable to a given bio-entity, such as gene, protein, pathway, operon, experiment, COG, and other. The system dynamically incorporates new sets of objects and their relations and integrates the following four complex orthogonal data types: Graphs, Tables/Histograms, Trees, and Sequences [14,15]. The problems of data integration are addressed via ontology-driven data mapping, multiple data annotation and heterogeneous data querying, also enabling integration of the user's data. The internal database schema is RDF-compatible [16]; that is, it stores biological data in the RDF-compatible format, the standard format of the Semantic Web [17]. The IntegromeDB web site supports RDF format by providing export of the Search Results web page in RDF format (at the top of the page; Figure 2) and also providing download of some parts of the database that describe organisms, pathways, synonyms, and interactions as the RDF formatted files http://integromedb.org/download_rdf.jsp.

The major architectural blocks of the system are a web-crawler, archive server, data integration server, internal database, and ontological mediator (Figures 1 and 1S1). The crawler automatically searches public databases, PubMed, and web resources for biological data and extracts them from texts and tables. The system accepts the user's data that can be integrated together with public data. The Data Integration Server accepts external data, semantically mapping them to the schema of the Internal Database, and injects the external data into the database. This allows IntegromeDB to tap into the Deep Web, the portion of the web that is concealed behind web forms. Some estimates have pegged the size of the Deep Web at up to 500 times larger than the Surface Web [18]. Briefly, the procedure to process the data behind web forms works as follows. Starting from an initial prediction of candidate keywords by simple ontological analysis of the text on the page (usually it is ether gene, protein, drug, pathway names), the form is tested and if it returns valid results, the common URL pattern of meaningful pages (for example 'http://www.ncbi.nlm.nih.gov/gene/?term=') is analyzed and further keywords will be extracted from the resulting pages. The iterative process is continued until either no new candidate keywords can be extracted or no more new web pages can be retrieved for a given resource.

The IntegromeDB content is available for more than 100,000 genes and proteins from 1252 organisms, more than 2 million experiments, 1000 pathways, 900,000 gene and protein records, and thousands of PubChem [19] records. Since many of the integrated resources are frequently updated, the IntegromeDB database is re-loaded on a quarterly basis. To ensure that the connections between external databases within IntegromeDB are as accurate as possible, an automated process checks for updates of outside databases, downloads files, and populates database tables.

### Querying IntegromeDB

Search on the IntegromeDB web page can be executed via keyword, wild card, or multiple word queries. Genes/proteins can be queried using either the gene name (the official HGNC [20] name, Entrez Gene [11], Ensembl [21]), Affymetrix identifier, current UniGene/UniProt [1] cluster identifier, or GenBank [2] accession number of a sequence (Refseq ID) associated with the gene through UniGene/UniProt (Figure 1D). We have assembled the largest collection of gene aliases available on the web by combining synonymous data from more than a thousand databases. IntegromeDB can therefore accept practically any known ID as input. To display data for a specific gene (Figures 1F, 2), the user must first select a gene from the search result list (Figure 1E). The Search results are divided into two groups: Exact Search "p53" and Relevant to "p53". The first group contains genes which names contain the query term (p53); the second group contains genes which were found in the same publications or the same database records with the query term.

One of the important features of IntegromeDB is the ability to simultaneously extract data for multiple genes in a batch, thus eliminating the need for laborious cross-referencing of data from external databases. The batch search is particularly useful for functional genomics studies, where it is necessary to regularly update annotations associated with genes/proteins being examined. For example, researchers interested in the mapped position or subcellular localization of a list of genes can extract these attributes and perform further analyses, assessing the enrichment of transcription factor binding sites or a certain functional attributes within clusters of genes. List of genes can be input as a text file via uploading on the server or by pasting it into a search box. The batch search is currently limited to 1000 genes.

The cornerstone of the IntegromeDB search engine is the IntegromeDB ontology and Categorization Engine (ICE) (Figure S1 in Additional file 1). The IntegromeDB ontology, which is described in detail in [13,14], integrates over a hundred OBO ontologies and consists of millions of terms organized as a directed acyclic graph (DAG), reflecting 'is-a', 'has-a', and 'part-of' relationships; for example, 'p53 is-a transcription factor'. Given a user query, ICE determines the nodes in the ontology that are most closely connected with a query. For example, for the 'p53' query, ICE first determines that p53 is a tumor suppressor, which is related to carcinogenesis and cancer pathogenesis. It also determines that p53 is a family of transcription factors related to cell signaling and playing role in the immune system and cell proliferation.

For each ontological term *A *and for each web page, or a document, *X*, integrated into the system, the relevance *RL *of the document *X *to the term *A*, is calculated as follows:

RL(X,A)=K1×PO(A)+K2×PP(X)+K3×PT(X)+K4×PR(X),

where *K1-K4 *- empirical coefficients (K1 = 0.2; K2 = 0.5; K3 = 0.4; K4 = 0.8); *PO *- frequency of a term *A *in the ontology which the term *A *belongs to; *PP *- frequency of a term *A *in the document *X*, this value corresponds to the *Lucene *keyword search score [22]; *PT *- frequency of the term *A *in different HTML tag fields of the web page (document) *X; PR *- PageRank [23] of the document *X*. In the process of executing the query containing the term *A*, ICE retrieves the top one thousand ranked documents.

**Figure 3:**
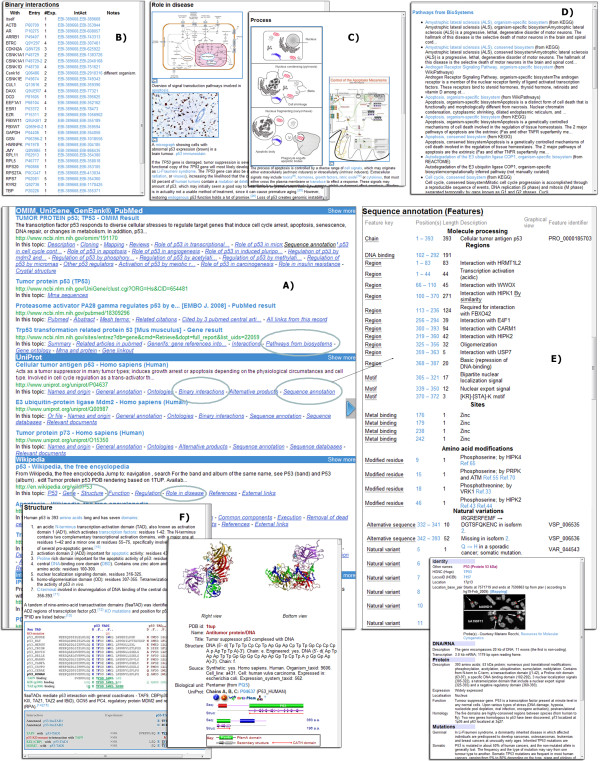
**Detailed view of the web data on the IntegromeDB report page. A) **Search results are grouped by data sources (UniProt, GenBank, PDB, *etc*.), and groups are separated by blue bands. In every group, data are presented according to their relevance to the search criteria. Rolling over the 'In this topic' item (in circles) shows an Instant Preview of the respective item, for example: **B) **Binary Interactions, **C) **Role in disease and Process, **D) **Pathways, **E) **Sequence Annotation, **F) **Structure.

**Figure 4:**
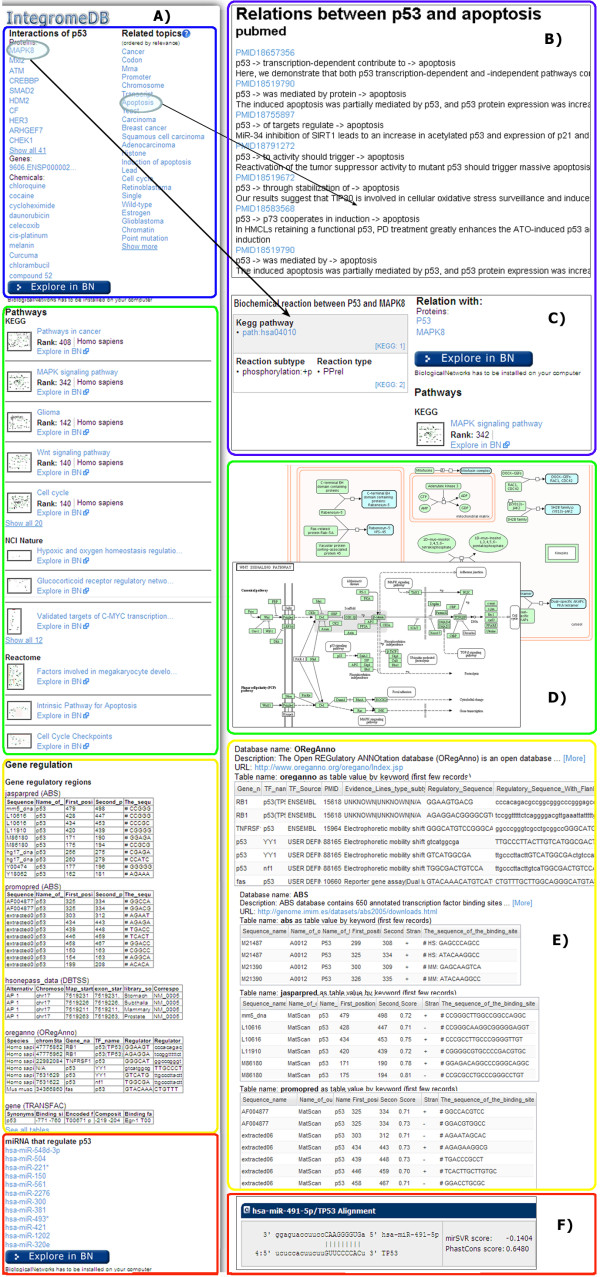
**Representation of table and relational data on the IntegromeDB report page. A) **Overview of the right panel of the report page. **B) **Relational data presented as topics, for example, for relations between p53 and apoptosis. **C) **Data on interactions and pathways, for example, the interaction between P53 and MAPK8 with detailed view of associated canonical pathways **(D). E) **Tables contain data on transcription factors and gene regulatory regions. **F) **Data on non-coding RNA.

**Figure 5:**
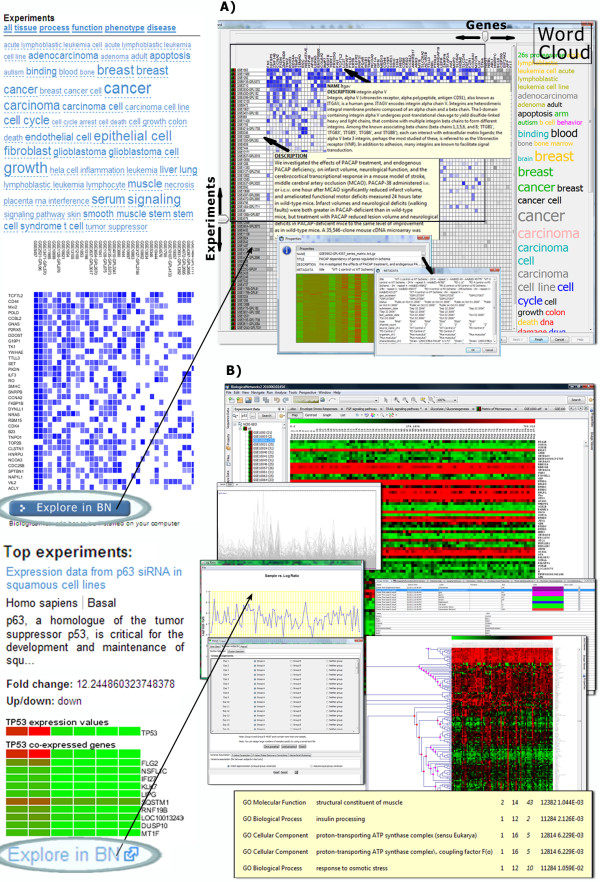
**Representation of experimental time-value data on the IntegromeDB report page. A) **Microarray experiments are clustered and represented as the multi-experiment matrix of the most often co-expressed genes with a query gene over a variety of tissues, cell types and diseases, which are represented as a cloud of words. **B) **The matrix and individual experiments can be further explored in BiologicalNetworks http://www.bioloigcalnetworks.org application by clicking on 'Explore in BN.'

Documents, or web pages, that are found to be relevant to the query gene/protein are further grouped together by data sources (Figure 3) and by similarity among documents. The similarity between two documents is defined as follows. Assume that a document is seen as a sequence of 'words', where a 'word' can be any combination of letters and digitals, representing biological information. It can be a simple word, like *'diabetes'*, or an abbreviation, like *'Leu'*, a *sequence *word 'ATGCTGGG' or 'MEPQSDVL', a number, or a combination of letters and numbers, like 'Hs.654481'. Further assume that for each word *i *a threshold frequency to be in the document is set at *b(i)*. Now, for each document, we can compute a vector in which the *i*-th component is set to 1, if the value of relative frequency of the word *i *in the document is greater than *b(i)*, or 0, otherwise. This binary vector is regarded as a fuzzy digital signature of the document and is built on the *descriptive set of words*. Vectors with 3 or less words above threshold are removed from further consideration. Low complexity words, such as 'ATGC', 'MEPQSDVL', or '0123456789', are considered as unique sets of symbols and are not considered in the vectors. The descriptive set of *N *words, which is defined for the whole database once it is updated, consists of the 'quality' words that cover nearly the maximum amount of documents thus that *N *is nearly minimal, where *N *is determined empirically. The 'quality' of a word is determined as relative stability of the corresponding component of the vector in similar documents. The threshold *b(i) *is chosen thus that the most of the documents have non-zero vectors. To estimate the similarity between two documents, the normalized Levenshtein distance [24] is used. Two documents are deemed similar if the difference between them does not exceed 10%. This rough estimate was obtained via assertion of the similarity between randomly chosen 300 documents by six researchers. Figures 4 and 5 show the documents (web pages) grouped by synonyms, sequences, pathways, regulatory and other types of data for the query 'p53'.

## Utility

### IntegromeDB web interface

IntegromeDB report page uses a two-dimensional newspaper-like layout (Figure 2) rather than as a search results page as in standard search engines (*e.g.*, Google). Data are grouped by data sources and similarity and sorted in each group by the relevance to the query gene/protein as is shown in Figures 2, 3, 4 and 5. The report page also provides a list of topics related to the query gene/protein (Figure 4). The links to the corresponding data sources are provided. Among data displayed for each gene/protein on the left panel of the report page (Figure 3) are aliases and external IDs, ontological terms, chromosome localization with links to NCBI and UCSC genome browsers, orthologs/homologs from multiple organisms, protein and genomic sequences, and related publications. On the right panel of the report page (Figures 4, 5) are shown interaction data and pathways (from KEGG [25], NCI [26], REACTOME [27], BioCARTA [28]), experiments (e.g., expression, metabolomics and proteomics data from GEO [29] and ArrayExpress [30]), miRNA data (from miRBase [31] and microRNA.org [32]), images (*e.g.*, protein structures from PDB [33] and Wikipedia [34]), and relative mRNA expression frequencies derived from various cell and tissue types (from descriptions/metadata of experiments in GEO and ArrayExpress).

For example, for the query 'p53', the report page (Figures 2, 3, 4 and 5) describes that p53 is involved in apoptosis, cancer, prostoglandin metabolism pathway, and MAPK, Wnt, cell cycle and other canonical pathways. Detailed information for each pathway, including genes, proteins, and small molecules involved in it is provided. For example, for prostoglandin metabolism pathway, the user can learn that it is involved in metabolizing lipids into prostaglandins and plays an important role in pain and inflammation; that the protein encoded by human PTGS1 gene is involved in the conversion of prostaglandin PGG2 into inflammation-causing prostaglandin PGH2; and aspirin has been shown to bind to the PTGS1 gene product (prostaglandin-endoperoxide synthase 1), blocking the ability of this enzyme to produce PGH2 and thereby reducing pain and inflammation.

#### Web Data

For each gene/protein, information retrieved from web pages is clustered by data sources (Figure 3). The user can scan the content of the web page by rolling over the respective term (e.g. 'Binary Interactions', 'Sequence Annotation', 'Structure') at the bottom of the snippet under *'In this topic'*. For example, for the 'p53' query, among the most relevant (valuable) resources were OMIM, UniProt, UniGene, GenBank, PubMed, Wikipedia, GeneCards, InterPro, p53.free.fr (mutation database), SYSTERS (protein families database), tp53.org. They were followed by (accessible via clicking the *'Show More Pages' *button) EMBL/EBI, MGD, STRING, WikiGenes, Genetic Atlas, CancerIndex, ProteinAtlas, KEGG, UCSC genome Browser, HAGR (Human Ageing Genomic Resource), SwissProt, PharmGKB and others.

#### Table data

In contrast to table data stored in listed and well-maintained databases, the tables published in the PubMed articles, as well as separate tables distributed across the web, are barely searchable by any search engine. Here, these data are integrated and become searchable using the same approach that is applied to the tables in the databases. Specifically, it concerns tabular data on gene regulatory regions, gene/protein interactions, and gene expression experiments.

To integrate the tables, IntegromeDB looks for web pages containing the HTML 'Table' tag and distinguishes relational tables (that are further parsed) from non-relational (that cannot be integrated), using a combination of in-house and statistical classifiers, e.g., calculating the ontology term in each column of the table; the classifiers have been trained on manually selected examples and use the information already integrated in IntegromeDB. If the columns containing significant percentage (empirically defined value) of the object IDs from IntegromeDB can be found, the table will be further processed; otherwise, filtered out. For example, a two-column table containing gene names and promoter sequences will be identified as a relational table as the percentage of ontologically defined objects in the first (gene names) column is high. Data from the other column will be considered as gene attributes if types of data can be defined, or named; that is, the column contains a data label in the first row. Thus, if in the example table the first cell of the second column contains the word 'promoter' and the other cells, DNA sequences, the postprocessor will understand that data in this column are promoter sequences. It will therefore scan the upstream region of the respective gene from the first column, and in case of finding the matching sequence, remembers its chromosomal localization in the global genomic coordinates and assigns to the gene the sequence in the corresponding cell as the attribute of the type *promoter_sequence*. If the sequence is not found, the postprocessor will assign the sequence as a new attribute with the name as provided in the first cell of the second column, and the type of the attribute will be *sequence*. Figure 4E shows gene regulatory data that was found for the 'p53'query from the databases provided information on gene regulatory regions and transcription factors.

Experimental tables containing, for example, microarray data, are clustered based on the Z-scores obtained using Fisher r-to-Z transformation of Pearson correlation coefficients calculating co-expression of the query gene with each gene in each experiment (data for each gene are averaged over multiple probes). The resulting co-expression matrix (Figure 5) highlights the experiments and genes (the intensity of blue correspond to the increasing value of the Pearson correlation coefficient above 0.75 and up to 1.0) co-expressed with the query gene (p53, in this case), and the word cloud shows a variety of conditions, tissues, and disease states found in the descriptions of these experiments. Visual inspection of the matrix allows detecting patterns of correlation across data sets and spot significantly strong co-expression profiles. Most often correlating genes (with the largest average Z-score over all experiments) and most related experiments (with the largest average Z-score over all genes) are located in the left-top corner of the matrix. For example, p53 was found to be overexpressed in cancers (breast, brain, bone marrow, squamous carcinoma, etc.), fibroblasts, astrocytes, and other cell types.

### Integration with other resources

One of the central aims of the IntegromeDB is to maintain cross-connectivity and integration with other public resources in a user-friendly manner. Therefore, we provide the programmatic access to our SQL database that is accessible via the following routes. First, the integrated content of IntegromeDB is available via the IntegromeDB API, which is implemented in Java. Through an XML-RPC service, API provides functions to access programmatically most of the features available in the IntegromeDB web interface, such as retrieving aliases, promoter sequences, or transcriptional regulators for a set of genes. Example code of using API and access to the XML-RPC service are available at http://integromedb.org/api.jsp ('API XML-RPC' tab). Second, if the external user/resource wants to visualize retrieved object(s) (interaction network of a protein, promoter region of a gene, microarray experiments for a set of genes, *etc*.) on his web resource using the BiologicalNetworks integrated research environment [13] he/she should use API access described at the http://integromedb.org/api.jsp 'API BiologicalNetworks' tab. Thus IntegromeDB/BiologicalNetworks maintains mutual cross-referencing with other web resources, that is not limited to simple text-based HTML links, but also enables partner websites to embed visualization of the BiologicalNetworks objects within their own web pages.

**Figure 6:**
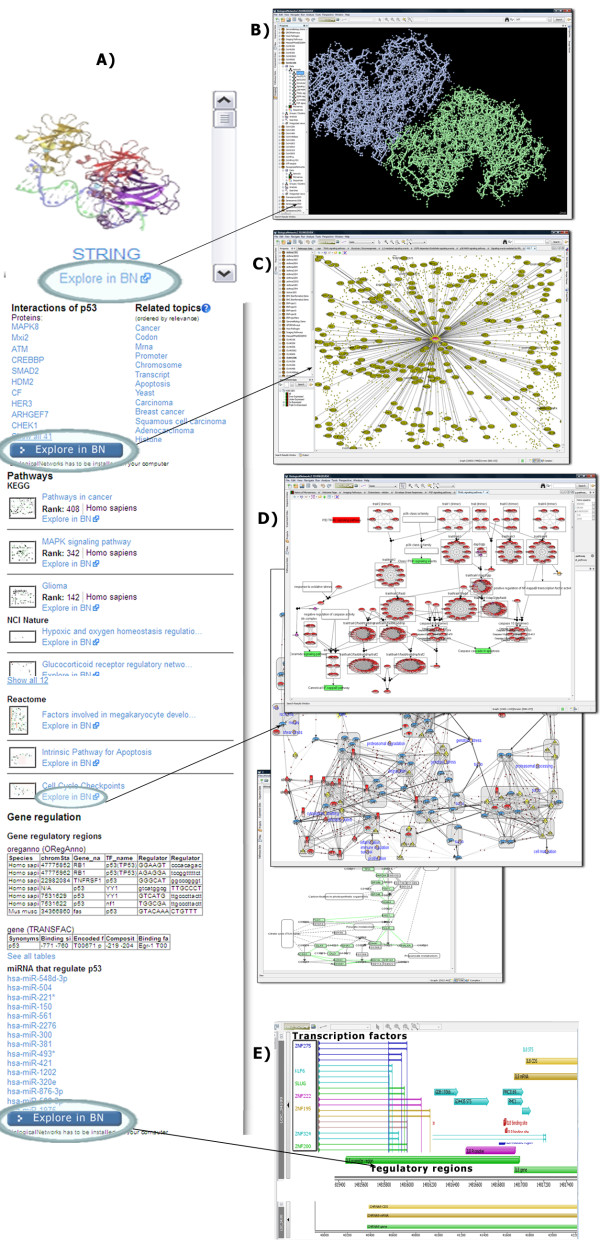
**Visualization and exploration of different types of biological information in BiologicalNetworks integrated research environment. A) **Overview of the right panel of the report page. **B) **Visualization of 3D protein structure. **C) **Visualization of protein-protein interaction networks. **D) **Visualization of pathways data. **E) **Visualization of data on gene regulatory regions.

Most of the data (pathways, networks, microarray experiments, sequences, etc.) returned by the IntegromeDB web site as a search results can be opened for detailed exploration and analysis in BiologicalNetworks. Figure 6 illustrates several examples of the interactions between IntegromeDB and BiologicalNetworks; in these scenarios, IntegromeDB is used for browsing large volumes of data, while BiologicalNetworks is used for exploring individual datasets in finer detail.

Partner websites or third-party software programs can choose to embed the entire IntegromeDB website into their own software. Thus, an IntegromeDB 'plugin' can be established at the BioGPS [6] portal, which provides 'plug-ins' through which the users can connect any number of external websites into freely configurable screen layouts.

## Discussion and Conclusions

Centralized and publicly available resources and search engines for integrated biological data are critical in enabling biologists to efficiently analyze genome-scale data. To meet this demand, we developed IntegromeDB, the general purpose unifying resource and search engine that can take the exploration of molecular biology data to the next level. IntegromeDB uses a hybrid approach to the Deep Web that combines elements of the crawl and federated search and data preprocessing approaches. It follows the trend established by other integrative resources, BioGPS [6] and Entrez [11], for example, that aim at presenting available information in a gene-centered manner. However, the technology behind IntegromeDB differs from that of other resources as it relies on data integration via automatic establishment of Object-Object Property relationships rather than on hyperlinks and manual curation. This approach is scalable in respect of how many data sources can be integrated (the number can be limited only by the hardware availability) and relatively inexpensive to develop and maintain as we deliberately keep human intervention at a minimum. However, as any automatic approach it has the drawback that unrelated data can be occasionally retrieved. With more usage of the site and more errors reported, the search algorithm will be improved. We encourage the users to report the errors and provide critique using the "Provide Feedback" button at the top right of the search results page (Figure 2).

To further enhance the ability of researchers to extract and manipulate the data, we will continue to provide the access to IntegromeDB through the API and data integration tools available in BiologicalNetworks making the most often used tools implemented as web services, thus eliminating the necessity to download BiologicalNetworks. We will also work on providing execution of SPARQL queries on the IntegromeDB data and create a SPARQL to SQL adaptor, using specifically designed dictionaries (that provide description of triples along with exact mappings onto tables).

We plan to continue extending the resource by adding new data sources and more experimental data and further developing the search engine's usability and speed. To improve the resource design and usability, we will rely on external experts (subject of funds availability) and we welcome and encourage community feedback that can be provided using the "Provide Feedback" button at the top right of the search results page. The speed of the presented search engine depends on both software and hardware; and while we will continue optimizing the search algorithms, we also plan to utilize a new hardware, specifically, a supercomputer Gordon that will become available at the San Diego Supercomputer Center in 2012.

## Availability and requirements

• **Project name: **IntegromeDB

• **Project home page: **http://www.integromedb.org

• **Operating system(s): **Platform independent. Tested on the Internet Explorer 8, FireFox 8, Google Chrome web browsers on Windows 2000/XP/Vista/7, Linux/Ubuntu/Redhat, and MacOSX OS.

• **Programming language: **Java

• **Other requirements: **Java 1.6 for running BiologicalNetworks program;

• **License: **GNU GPL version 3

• **Any restrictions to use by non-academics: **none

## Competing interests

The authors declare that they have no competing interests.

## Authors' contributions

SK, YD and MB designed and implemented the system and performed major programming work, JP contributed to the system concept, MB and JP wrote the manuscript. This work was coordinated by MB. All authors read and approved the final manuscript.

## Supplementary Material

Additional file 1 **Components of the IntegromeDB architecture and Search algorithm**. Major components of the IntegromeDB search engine. Click here for file
