# Supplementary material for: IntegromeDB: an integrated system and biological search engine
Source: BMC Genomics. 2012 Jan 19;13:35. doi: 10.1186/1471-2164-13-35 (PMC3293773; doi:10.1186/1471-2164-13-35)
Supplement: Additional file 1 — Components of the IntegromeDB architecture and Search algorithm. Major components of the IntegromeDB search engine. [file 1471-2164-13-35-S1.DOC]

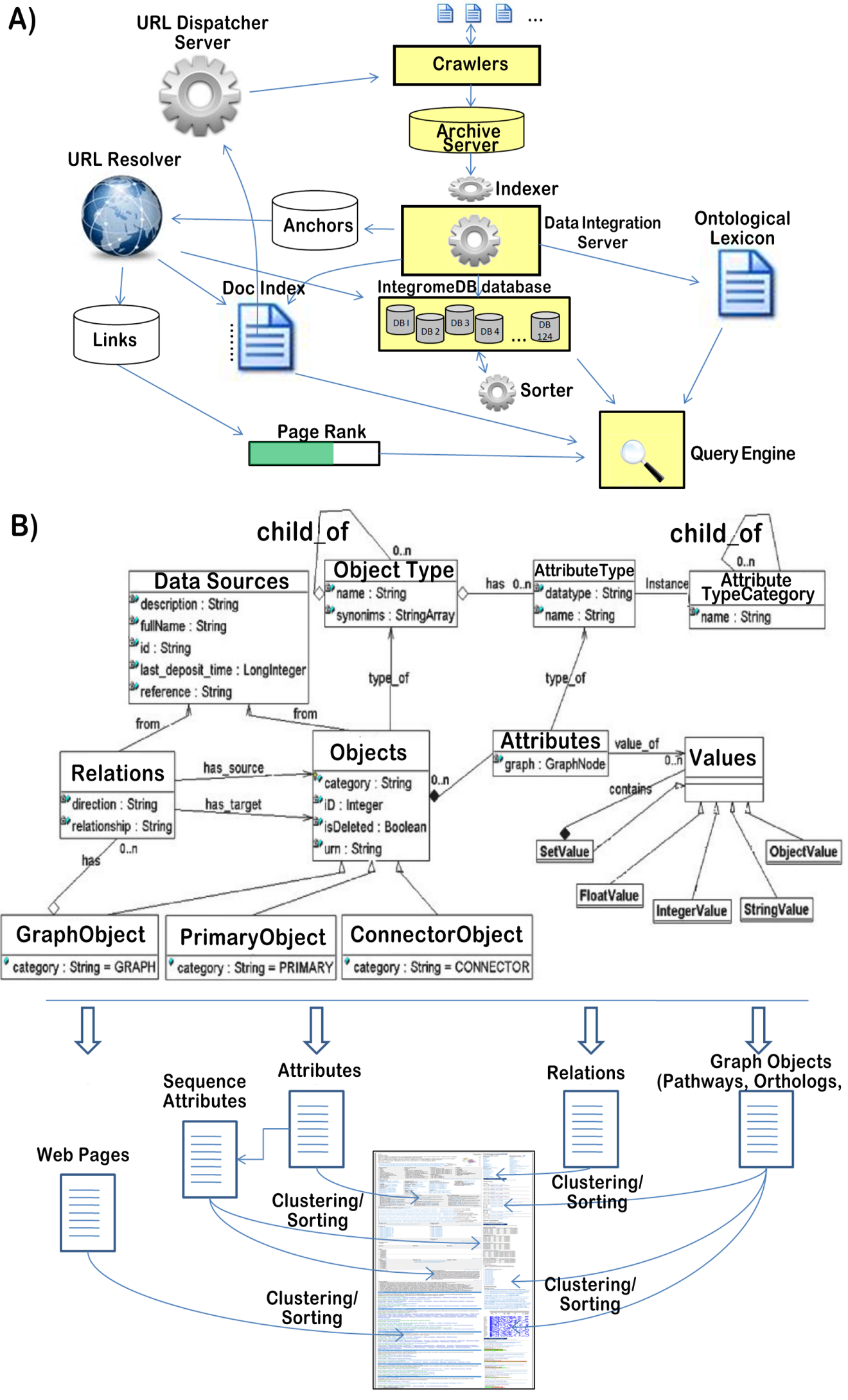


**Figure S1. Components of the IntegromeDB architecture and Search algorithm. A. Major components of the IntegromeDB search engine.** *Crawlers*: There are several distributed crawlers that parse the pages and extract links and keywords. *URL Dispatcher Server* provides to the crawlers a list of URLs to scan. *Archive Server*: The crawlers send collected data to the archive server. It compresses the pages and places them in the repository. Each page is stored with an identifier, docID. *Indexer* indexes pages using *Lucene* indexing technology. The indexer decompresses documents, converts them into sets of words called "hits", and creates a list of URLs on each page. A hit contains the following information: the word, its position in the document, font size, capitalization. *Data Integration Server* extracts data from web sites and populates the IntegromeDB schema. *Anchors*: The bank of anchors is created by the indexer; it contains internal links and text associated with each link. *URL Resolver* takes the contents of anchors, converts relative URLs into absolute addresses and finds docID or creates a new one. It also builds an index of documents and a database of links. *Doc Index* contains the text relative to each URL. *Links*: The database of links associates each link with docID (and thus with a real document on the Web). *PageRank*: The software uses the database of links to define the PageRank of each page. *Sorter* interacts with the depository, includes documents classified by docIDs and creates an inverted list sorted by wordIDs. *Ontological Lexicon*: A software takes the list provided by the sorter (classified by wordIDs), and also includes the lexicon created by the indexer (the sets of keywords in each page), and produces a new lexicon to the searcher. **B. Query Engine algorithm.** The query algorithm runs on a web server. It parses the input query string, converts words into wordIDs and scans all the documents and database objects that match all the search terms. Then, using the Ontological Lexicon built on the BioNets Ontology in combination with the index classified by wordIDs, the algorithm computes the ranks of each object and each document (using formula from the Main text that takes into account the PageRank). Further, the algorithm sorts the objects and documents and returns the top *k* documents and the top *k* objects (*k*=1000). For each returned object and document, the algorithm extracts their properties; for the object, Object properties, Object relations (e.g. interactions), and Graph Objects (e.g. pathways); for the document, Page Rank, Lucene keyword score, and the ontological keyword score. Finally, the properties are sorted, clustered and represented on the web page (see Figure 2 in the main text).
